# The therapeutic potential of a series of orally bioavailable anti-angiogenic microtubule disruptors as therapy for hormone-independent prostate and breast cancers

**DOI:** 10.1038/sj.bjc.6604100

**Published:** 2007-11-20

**Authors:** S P Newman, P A Foster, Y T Ho, J M Day, B Raobaikady, P G Kasprzyk, M P Leese, B V L Potter, M J Reed, A Purohit

**Affiliations:** 1 Endocrinology and Metabolic Medicine and Sterix Ltd, Faculty of Medicine, Imperial College London, St Mary's Hospital, London W2 1NY, UK; 2 IPSEN-Biomeasure, 27 Maple Street, Milford, MA, USA; 3 Medicinal Chemistry and Sterix Ltd, Department of Pharmacy and Pharmacology, University of Bath, Claverton Down, Bath BA2 7AY, UK

**Keywords:** microtubule, breast, prostate, angiogenesis

## Abstract

Therapies for hormone-independent prostate and breast cancer are limited, with the effectiveness of the taxanes compromised by toxicity, lack of oral bioavailability and drug resistance. This study aims to identify and characterise new microtubule disruptors, which may have improved efficacy relative to the taxanes in hormone-independent cancer. 2-Methoxy-3*-O-*sulphamoyl-17*β*-cyanomethyl-oestra-1,3,5(10)-triene (STX641), 2-methoxy-3-hydroxy-17*β*-cyanomethyl-oestra-1,3,5(10)-triene (STX640) and 2-methoxyoestradiol-3,17-*O*,*O*-bis-sulphamate (STX140) were all potent inhibitors of cell proliferation in a panel of prostate and breast cancer cell lines. STX641 and STX640 significantly inhibited tumour growth in the MDA-MB-231 xenograft model. STX641 inhibited both *in vitro* and *in vivo* angiogenesis. Despite good *in vivo* activity, STX641 was not as potent *in vivo* as STX140. Therefore, STX140 was evaluated in the prostate hormone-independent PC-3 xenograft model. STX140 had superior efficacy to docetaxel, 2-MeOE2 and bevacizumab. In contrast to vinorelbine, no significant toxicity was observed. Furthermore, STX140 could be dosed daily over a 60-day period leading to tumour regression and complete responses, which were maintained after the cessation of dosing. This study demonstrates that STX641 and STX140 have considerable potential for the treatment of hormone-independent breast and prostate cancer. In contrast to the taxanes, STX140 can be dosed orally, with no toxicity being observed even after prolonged daily dosing.

Prostate cancers initially respond to androgen ablation therapy; however, most will progress to hormone-independent disease and subsequent advanced metastatic cancer. Few efficacious treatment options are available for advanced prostate cancer, although recent studies have shown the taxanes to offer modest clinical benefit (Berry and Eisenberger, 2005). While much progress has been made in the treatment of ER-positive breast cancer, such as the recent introduction of aromatase inhibitors, fewer options are available for hormone-independent breast cancer. The taxanes, paclitaxel (taxol) and docetaxel (taxotere), are routinely used in late-stage metastatic breast cancer and have also been successfully trialled in the adjuvant and neoadjuvant setting for early breast cancer (Ring and Ellis, 2005). Although initially responsive, many tumours quickly become resistant to taxane therapy and the disease progresses. *In vivo*, this resistance is largely due to the expression of the *P*-glycoprotein pump (Fojo and Menefee, 2005) and/or the overexpression of the *β* type III tubulin isoform (Burkhart *et al*, 2001). In addition, the toxicity of the taxanes limits their use. Standard therapies with taxanes are based around a schedule of one dose every 3 weeks, and before i.v. taxane therapy can be administered the patients need to be premedicated with dexamethasone to prevent hypersensitivity reactions (Marchetti *et al*, 2002; Crown *et al*, 2004). Thus, there is an unmet need, in both hormone-independent prostate and breast cancers, to develop a new series of compounds with the anti-tumour properties of the taxanes but with an improved clinical profile.

Targeting tumour angiogenesis is a major focus of anti-cancer research; however, to date, few single-agent anti-angiogenic agents have entered clinical use (Eskens, 2004). There has been success in combining anti-angiogenic agents with conventional chemotherapy, such as the recent successful trial of bevacizumab with 5-fluorouracil-based chemotherapy for metastatic colon cancer (Hurwitz *et al*, 2004). Furthermore, the potential anti-angiogenic activity of many conventional chemotherapy agents is being explored by the use of low-dose metronomic dosing, in an effort to combine their anti-tumour activity with an anti-angiogenic activity (Vacca *et al*, 1999; Hanahan *et al*, 2000; Bertolini *et al*, 2003). However, such dosing regimes, while likely to be anti-angiogenic, will result in the drug being used at a suboptimal dose for direct anti-tumour effects. Therefore, development of agents, which target the tumour cells directly, target the tumours blood supply and can be administered regularly should prove to be a productive area of research (Eskens, 2004).

One such potential agent is the natural metabolite of oestradiol, 2-methoxyoestradiol (2-MeOE2), which over the last decade has shown promise both *in vitro* and *in vivo* (Pribluda *et al*, 2000; Dingli *et al*, 2002; Lakhani *et al*, 2003). 2-Methoxyoestradiol is an anti-angiogenic and anti-proliferative agent *in vitro* (Zhu and Conney, 1998; Shang *et al*, 2001). 2-Methoxyoestradiol inhibits the *in vivo* growth of xenografts derived from human MDA-MB-435 breast cancer cells, MethA sarcomas, B16 melanomas and the multiple myeloma cell line KAS-6/1 in nude mice (Fotsis *et al*, 1994; Klauber *et al*, 1997; Dingli *et al*, 2002). However, the *in vivo* efficacy of 2-MeOE2 is poor, with comparatively high oral (p.o.) or intraperitoneal (i.p.) doses of 75 and 150 mg kg^−1^ per day, respectively, being required to reduce the growth of melanoma or myeloma tumours in rodent models (Klauber *et al*, 1997; Dingli *et al*, 2002). In phase I trials, a clinical benefit was shown only in two patients who were receiving 1600–3200 mg day^−1^ 2-MeOE2 orally. The trial was stopped early due to extremely low plasma concentrations of 2-MeOE2 even at 3000 mg day^−1^ (Dahut *et al*, 2006). One possible explanation is provided by the observation that 2-MeOE2 is oxidised *in vitro* by tumour cell lines, which express 17*β*-HSD type 2 (Purohit *et al*, 2000; Liu *et al*, 2005). The gastrointestinal tract expresses 17*β*-HSD type 2 (Sano *et al*, 2001), and this may inactivate 2-MeOE2 before it enters the blood stream. This is supported by data from a recent phase I trial in which a daily oral dose of 1000 mg 2-MeOE2 was given to 24 patients with advanced metastatic breast cancer. Metabolism studies showed that 80–95% of the 2-MeOE2 was oxidised to 2-methoxyoestrone (2-MeOE1), and furthermore, 80–90% of both 2-MeOE2 and 2-MeOE1 were present as glucuronide or sulphate conjugates. Additional evidence for the poor oral bioavailability of 2-MeOE2 comes from the work of Ireson *et al* (2004), who demonstrated that 2-MeOE2 could not be detected in the plasma of rats 1 h after administration of a single oral dose of 2-MeOE2 (10 mg kg^−1^). The problems of poor bioavailability and rapid metabolism associated with this drug may be overcome by synthesising analogues resistant to conjugative and metabolic inactivation (Leese *et al*, 2005b). One such compound is 2-methoxyoestradiol-3,17-*O,O*-bis-sulphamate (STX140), whose potential anti-angiogenic and anti-tumour activities have been well documented (Day *et al*, 2003; Ho *et al*, 2003; Newman *et al*, 2004; Wood *et al*, 2004; Raobaikady *et al*, 2005; Utsumi *et al*, 2005; Leese *et al*, 2006).

In the present study, we characterise the recently developed (Utsumi *et al*, 2005) C-17-substituted compound, 2-methoxy-3*-O-*sulphamoyl-17*β*-cyanomethyl-oestra-1,3,5(10)-triene (STX641) and the corresponding nonsulphamoylated compound, 2-methoxy-3-hydroxy-17*β*-cyanomethyl-oestra-1,3,5(10)-triene (STX640). Furthermore, we assess their *in vivo* efficacy in hormone-independent cancer and compare them with STX140.

## MATERIALS AND METHODS

### Drug synthesis

**Figure 1:**
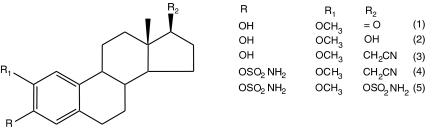
Structures: compound 1, 2-methoxyoestrone (2-MeOE1); compound 2, 2-methoxyoestradiol (2-MeOE2); compound 3, 2-methoxy-3-hydroxy-17*β*-cyanomethyl-oestra-1,3,5(10)-triene (STX640); compound 4, 2-methoxy-3*-O-*sulphamoyl-17*β*-cyanomethyl-oestra-1,3,5(10)-triene (STX641); and compound 5, 2-methoxyoestradiol-3,17-*O*,*O*-bis-sulphamate (STX140).

2-Methoxyoestrone (2-MeOE1, Figure 1, compound 1) and 2-methoxyoestradiol (2-MeOE2, Figure 1, compound 2) were synthesised by literature routes (Leese *et al*, 2005a). 2-Methoxyoestradiol-3,17-*O,O*-bis-sulphamate (STX140; Figure 1, compound 5) was synthesised by reaction of 2-MeOE2 with sulphamoyl chloride in dimethylacetamide (DMA) (Leese *et al*, 2006). The 17-cyanomethyl compounds 3 and 4 were elaborated from 2-methoxy-3-*O*-benzyloestrone by Wadsworth–Emmons reaction followed by hydrogenolysis to afford 3. Sulphamoylation of compound 3 with sulphamoyl chloride in DMA yielded the 3-*O*-sulphamate derivative compound 4. Full details of these syntheses will be reported elsewhere.

### Cell culture

Human umbilical vein endothelial cells (HUVECs) were obtained from TCS Cellworks (Claydon, UK) and maintained in large vessel endothelial medium supplemented with basic fibroblast growth factor / heparin, epidermal growth factor and cortisol in the presence of amphoteracin/gentomycin (TCS Cellworks). Human adult dermal fibroblasts were obtained from TCS Cellworks and maintained in fibroblast growth medium (TCS Cellworks) with the same supplements as used in the HUVEC media. MDA-MB-231 (breast ER−ve), PC-3 (prostate AR−ve), MCF-7 (breast ER+ve), A2780 (ovarian ER−ve) and LNCaP (prostate AR+ve) cancer cells were obtained from the American Type Culture Collection (LGC Promochem, Teddington, UK) and maintained in Dulbecco's minimal essential medium containing phenol red, supplemented with 10% fetal calf serum and antibiotics (Sigma, Poole, UK). All cells were cultured at 37°C under 5% CO_2_ in a humidified incubator. HUVECs and dermal fibroblasts were used up to passage 10.

To ascertain the IC_50_ values 5000–10 000 cells, in their appropriate growth medium, were added to each well of a 96-well microtitre plate (Falcon; BD Biosciences, Cowley, UK). Plates were incubated for 4–5 h at 37°C in a 5% CO_2_ humidified atmosphere before addition of compounds at a final concentration of 0.1 nM to 10 *μ*M. All compounds were dissolved at 10^−2^ M in tetrahydrofuran (THF) with a final concentration of THF of 0.1% or less. Cells were grown in the absence or presence of the compounds for 5 days. At the end of this period, MTS (20 *μ*l per well; Promega, Southampton, UK) was added and incubated for a further 2 h. Absorbance was recorded at 490 nM with a 96-well plate reader (FLUOSTAR; BMG, Aylesbury, UK).

### Reversibility studies

Cells were plated at 60–70% confluency in T-25 flasks (Triple Red, Thame, UK), and after 24 h, they were treated with 0.5 *μ*M of the relevant compound. Control cells were treated with THF vehicle only. After 3 days, the medium was aspirated and the cell monolayers washed thoroughly. The number of cells in three flasks for each treatment was determined by Coulter counting. Of the remaining six flasks for each initial treatment, three were treated with 0.5 *μ*M of the appropriate compound, and three with vehicle alone. After a further 3 days, the remaining cells were counted using a Coulter Counter.

### Cell cycle analysis

Cells were plated at 60–70% confluency in T-25 flasks. After 24 h, they were treated with 0.5 *μ*M of compound for a further 24 h. Control cells were untreated or treated with THF vehicle only. To harvest cells for flow cytometric DNA analysis, cells were washed with PBS before being trypsinised (0.25% trypsin, 0.05% EDTA). Media containing nonadherent cells were also collected and pooled with the trypsinised cells. The cells and PBS washings were pelleted by centrifugation at 1500 r.p.m., washed twice with PBS, fixed in cold 70% ethanol, treated with 100 *μ*g ml^−1^ RNase for 5 min, stained with 50 *μ*g ml^−1^ propidium iodide and analysed using a flow cytometer (FACScan; Becton Dickinson, Cowley, UK).

### Apoptosis analysis

Cells were plated at 60–70% confluency in T-25 flasks. After 24 h, they were treated with 0.5 *μ*M of compound. Control cells were untreated or treated with THF vehicle only. To harvest cells for flow cytometric analysis, cells were washed with PBS before being trypsinised (0.25% trypsin, 0.05% EDTA). Any media containing nonadherent cells were also collected and pooled with the trypsinised cells. The cells and washings were pelleted by centrifugation at 1500 r.p.m., washed twice with PBS and re-suspended in binding buffer (10 mM HEPES/NaOH pH 7.4, 140 mM NaCl, 2.5 mM CaCl_2_) at 1 × 10^6^ cells ml^−1^. Cells were then stained with fluorescein-conjugated Annexin V (BD Biosciences) antibody and propidium iodide (5 *μ*g ml^−1^) before flow cytometric analysis. Apoptotic cells are defined as cells positive for Annexin V and negative for propidium iodide.

### Immunoblotting

T-25 flasks seeded at approximately 2.5 × 10^5^ cells per flask in 5 ml of cell-specific media were incubated at 37°C, 5% CO_2_ in a humidified incubator. After 24 h, the compounds were added and the cells incubated for 24, 48 or 72 h. Protein was prepared from treated cells using RIPA lysis buffer (Sigma). Equal amounts of protein were loaded in all wells. Proteins were separated by electrophoresis through a 4–12% NuPAGE Bis-Tris gel (Invitrogen, Paisley, UK) and subsequently transferred to Hybond-P membrane (Amersham Biosciences, Little Chalfont, UK). Detection was carried out using an anti-human cyclin B1 (sc-752) (Santa Cruz Biotechnology Inc., Santa Cruz, CA, USA) primary antibody.

### Tubulin polymerisation

The effects of test compounds (STX641, STX640 and STX140) on *in vitro* polymerisation of purified bovine brain tubulin (Cytoskeleton, Denver, CO, USA) were measured by turbidometry. Tubulin (1 mg ml^−1^) in MES buffer (0.1 M MES pH 6.5, 0.5 mM MgCl_2_, 1 M monosodium glutamate and 1 mM GTP) was incubated with or without test compounds (10 *μ*M, 1% v/v ethanol) for 5 min at 37°C. Tubulin assembly was stimulated by adding paclitaxel (10 *μ*M). The change in the absorbance was continuously monitored at 350 nm for 15 min at 37°C. Because the amount of tubulin polymerised is directly proportional to the area under the curve (AUC), it was used to determine the extent of tubulin polymerisation by different test compounds. The AUC of paclitaxel alone, with a maximal extent of polymerisation, was set to 100% polymerisation.

### Angiogenesis assay

The effect of the compounds on *in vitro* vessel formation was assessed using an angiogenesis kit (TCS Cellworks). For this assay, endothelial cells were cultured in a 24-well plate within a matrix of human diploid fibroblasts of dermal origin in optimised medium supplied by TCS Cellworks. The co-cultured cells were incubated throughout the experiment at 37°C under 5% CO_2_ in a humidified incubator. On day 1, the culture medium was removed and replaced with medium containing the compounds under investigation. On days 4, 7 and 9, the medium was replaced with fresh medium containing the compounds. Each compound was tested in triplicate. On day 11, the cells were washed (in PBS) and 70% ethanol (1 ml) was added to each well for 30 min to fix the cells. After fixation, the cells were washed with blocking buffer (1 ml, PBS+1% BSA) and stained for CD31 in accordance with the manufacturer's instructions (TCS Cellworks).

The extent of vessel formation was then quantified using a variation of a previously described technique (Newman *et al*, 2004). Briefly, using a high-resolution transmissive scanner (ScanMaker 9800; Microtek, Willich, Germany), each well was scanned and saved as a TIF (Tagged Image Format) file in Photoshop (Adobe, San Jose, CA, USA). The image was then converted to a black and white image using the photocopy filter in Photoshop (2 × , 10 Detail, 25 Darkness) and saved as an uncompressed TIF file. The files were transferred to the AngioSys software (TCS Cellworks), all background and nontubule-like structures were removed using the erode (1 ×) and clean (100 pixels) functions, and the number of pixels representing vessels was counted. This technique was validated (data not shown) against the quantification techniques as described previously (Newman *et al*, 2004).

### Xenograft models

Female MF-1 nu/nu mice were injected subcutaneously (s.c.) in the flank with 2 × 10^6^ MDA-MB-231 cells. Twice weekly i.p. administration of STX641 (40 mg kg^−1^) and STX640 (40 mg kg^−1^) was compared with daily oral dosing of STX641 (40 mg kg^−1^). Dosing was performed for 28 days after which animals were euthanised and the tumours taken for histological and IHC studies.

Male MF-1 nu/nu mice were injected s.c. in the flank with 2 × 10^6^ PC-3 cells. Daily oral dosing of STX140 (20 mg kg^−1^) was compared with 2-MeOE2 100 mg kg^−1^ (daily, p.o.), bevacizumab 5 mg kg^−1^ (q3d × 4, i.p.), taxotere 30 mg kg^−1^ (qwk × 3, i.v.) and vinorelbine 80 mg kg^−1^ (qwk × 3, p.o.). At the end of the initial 28-day study, the decision was taken to extend the dosing in STX140 arm for a further 32 days due to lack of toxicity and promising efficacy being observed.

In both studies, animal weights and tumour measurements were regularly taken using electronic callipers. Tumour volume (V), in mm^3^, was determined using the following equation: length × width^2^/2 (*l* × *w*^2^/2).

### Immunohistochemistry

Hematoxylin–eosin (H&E) staining and von Willebrand's factor IHC were performed on paraffin-embedded MDA-MB-231 tumour sections cut at 6 μm. After sectioning, rehydration and antigen retrieval steps, von Willebrand's factor antibody (1 : 800, Abcam, Cambridge, UK) was applied to the section for 1 h at RT, followed by a goat polyclonal secondary antibody conjugated to FITC (30 min at RT). Sections were then mounted and viewed under a light or fluorescence microscope.

### Statistics

All *in vitro* experiments were carried out in triplicate, and data presented are representative of one of three such experiments. All errors shown are the mean±s.d. Student's *t*-test was used to assess the significance of the differences in cell proliferation *in vitro*. For xenograft data, one-way ANOVA followed by a Bonferroni's multiple comparison test was performed to determine statistical significance on most data sets. Where only two groups are compared, a Student's *t*-test was applied. All values are represented as the mean±standard errors of the mean (s.e.m.). Data generated in these studies were normally distributed as assessed by the method of Kolmogorov and Smirnov. Statistics were calculated using Prism 3 for Mac (GraphPad Software Inc., San Diego, CA, USA).

## RESULTS

### Cell proliferation assays

The ability of STX641 and the corresponding nonsulphamoylated compound STX640 to inhibit the proliferation of a panel of cell lines was examined over a 4-day period (Table 1). The replacement of the *O*-sulphamate group at the 17 position of STX140 with a cyanomethyl group, to give STX641, increased *in vitro* potency by two- to five-fold in the tumour cell lines tested. Removal of the 3-*O*-sulphamate group of STX641 to generate STX640 led to a decrease in activity. The greatest decrease in potency was seen in the PC-3 AR−ve prostate cell line, where removal of the 3-*O*-sulphamate moiety caused over an eightfold decrease in potency (IC_50_ values; 50 *vs* 430 nM). Inhibition of HUVEC proliferation was used to assess the potential anti-angiogenic activity of these compounds. All three compounds were potent inhibitors of HUVEC proliferation with IC_50_ values of <50 nM, thus indicating that these compounds may be potent inhibitors of angiogenesis.

In contrast to their effects on cancer cells and HUVECs, human adult dermal fibroblasts were relatively resistant to the anti-proliferative effects of STX641, STX640 and STX140 with IC_50_ values in excess of 5 *μ*M (data not shown).

### Reversibility studies

**Figure 2:**
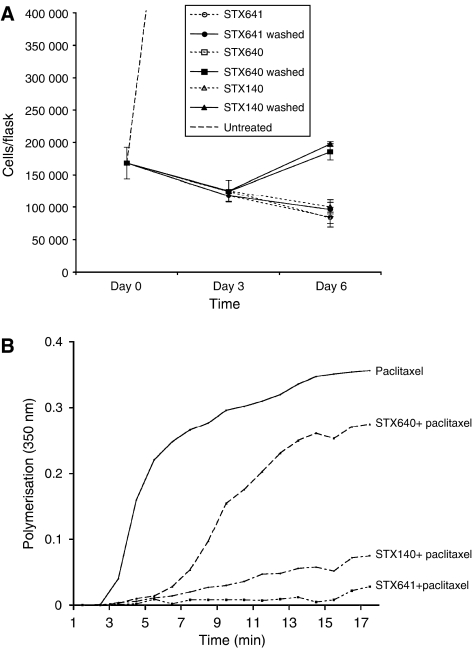
STX641, STX640 and STX140 are irreversible inhibitors of proliferation and disrupt microtubule dynamics. (**A**) PC-3 cells were exposed to compounds as indicated (0.5 *μ*M) for 3 days, and cell numbers were determined by Coulter counting. Cells were washed thoroughly to remove compounds, and cell numbers determined after a further 3 days: compound-free incubation (solid lines) or continued compound treatment (dashed lines). Values are means of triplicate determinations; bars, s.d. (**B**) *In vitro* tubulin assembly was measured by turbidity at 350 nm. Tubulin was incubated with or without test compounds (10 *μ*M) for 5 min at 37°C. Tubulin assembly was stimulated by adding paclitaxel (10 *μ*M). The change in the absorbance was continuously monitored at 350 nm for 15 min at 37°C. The AUC was used to determine the extent of tubulin polymerisation by different test compounds. The AUC of paclitaxel alone, with a maximal extent of polymerisation, was set to 100% polymerisation. The AUC for the test compounds was STX640, 63%; STX641, 3.4%; and STX140, 12.2%.

To assess whether the growth inhibitory effects seen were attributable to irreversible inhibition of cell proliferation or to delayed cell death, PC-3 cells were exposed to 0.5 *μ*M STX641, STX640 or STX140 for 3 days. The cells were then washed to remove the compound and cultured for a further 3 days in the absence of compound. In the prostate PC-3 cell line (Figure 2A), there was an increase in cell number following the removal of STX640 and STX140, but the rate of growth was still decreased compared to untreated cells. However, STX641 continued to cause a decline in the cell number, indicating that the cells were irreversibly committed to cell death. Similar data have been previously shown for STX140 in the hormone-independent breast cancer cell line, MDA-MB-231 (Raobaikady *et al*, 2005).

### Tubulin polymerisation

The inhibition of paclitaxel-stimulated tubulin polymerisation *in vitro* is commonly observed with many microtubule destabilising agents, such as colchicine and the vinca alkaloids. Previously, it has been shown that the sulphamoylated oestrone derivative, 2-methoxyoestrone-3-*O*-sulphamate, inhibited the paclitaxel-stimulated tubulin polymerisation, as measured by changes in turbidity (MacCarthy-Morrogh *et al*, 2000). In this study, both STX641 and STX140 significantly inhibited paclitaxel-stimulated tubulin polymerisation, with STX641 being the most potent inhibitor (96.6% inhibition *vs* 87.8% inhibition, Figure 2B). The nonsulphamoylated derivative, STX640, had some inhibitory activity (37% inhibition), in contrast to 2-MeOE2, which was previously shown to be inactive in this assay (MacCarthy-Morrogh *et al*, 2000). The vehicle alone, ethanol, had no effect on the paclitaxel-stimulated polymerisation of tubulin (data not shown).

### Cell cycle analysis

**Figure 3:**
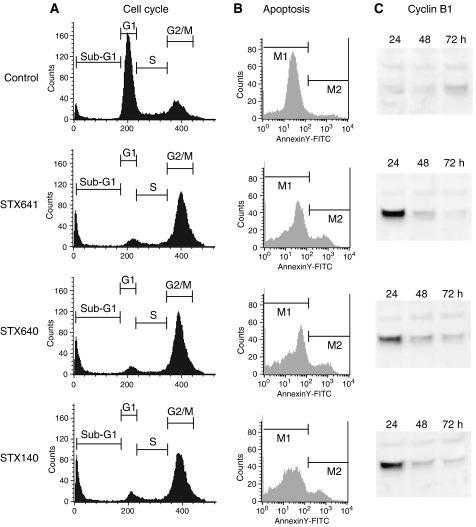
STX641, STX640 and STX140 cause cell cycle and subsequent apoptosis in PC-3 cells. (**A**) PC-3 cells were treated with 0.5 *μ*M compound for 24 h, before being fixed and stained with propidium iodide and analysed using a flow cytometer and CellQuest Pro software. The plots represent one of at least three representative experiments. (**B**) To identify cells undergoing apoptosis, PC-3 cells were treated with compounds (0.5 *μ*M) as indicated for 72 h. The cells were then harvested and stained with fluorescein-conjugated Annexin V antibody and propidium iodide before analysis using flow cytometer and CellQuest Pro software. The cells undergoing apoptosis are in M_2_ region of the histogram. The percentage of cells undergoing apoptosis is control, 4%; STX640, 16%; STX641, 16%; and STX140, 14%. The plots represent one of at least three representative experiments. (**C**) Immunoblot showing induction of cyclin B1. PC-3 cells were treated with compound (0.5 *μ*M) for 24 h, and total cell protein extracted and immunoblotted for cyclin B1.

It has been demonstrated that sulphamoylated derivatives of 2-MeOE1 and 2-MeOE2 are able to induce cell cycle arrest in MCF-7, ZR-75-1, CA51 and CAMA1 cells (MacCarthy-Morrogh *et al*, 2000). In this study, we examined the effect of C-17 modifications on cell cycle arrest in the hormone-independent PC-3 prostate cell line. In untreated PC-3 cells, approximately 60% of the cells are in G_1_ and 22% are in G_2_/M (Figure 3A). After treatment with 0.5 *μ*M STX641 for 24 h, 70% of PC-3 cells entered G_2_/M arrest, with only 6% in G_1_, which is almost the same distribution observed following 0.5 *μ*M STX140 treatment of PC-3 cells for 24 h (Figure 3A). The nonsulphamoylated compound, STX640 (0.5 *μ*M), also potently induced cell cycle arrest with 71% of PC-3 cells being in G_2_/M after 24 h and 6% in G_1_.

### Apoptosis

Cells exiting cell cycle arrest either continue through the cell cycle or undergo apoptosis. In this study, the capacity of STX641, STX640 and STX140 to induce apoptosis in the hormone-independent PC-3 prostate cell line was examined by looking at Annexin V flip out. Apoptosing cells are detected in the M_2_ region of the histogram in Figure 3B. All compounds induced significant (*P*<0.001) apoptosis after 72 h with respect to vehicle-treated control in PC-3 cells. Quantification of the histograms revealed no significant difference between the compounds. The data presented here compliment that of Raobaikady *et al* (2005), which demonstrated that STX140 induces apoptosis in the hormone-independent breast cancer line, MDA-MB-231.

The timing of the cell cycle arrest and subsequent apoptosis was further confirmed by immunoblotting for the cell cycle protein cyclin B1 in PC-3 cells. The induction of cyclin B1 by all three compounds coincided with the observed cell cycle arrest (Figure 3C). Once cyclin B1 levels start to decrease (48 h onwards), the cells undergo apoptosis (Figure 3B).

### Inhibition of *in vitro* angiogenesis

As these compounds are potent inhibitors of endothelial cell proliferation (Table 1 and Newman *et al*, 2004), their ability to inhibit *in vitro* angiogenesis was assessed. In this study, a co-culture model was used in which endothelial cells are co-cultured with fibroblasts in a specially formulated medium. The pro-angiogenic factor VEGF (2 ng ml^−1^) was used to further stimulate vessel formation in this assay, and the compounds' capacity to inhibit the VEGF-stimulated angiogenesis was assessed.

**Figure 4:**
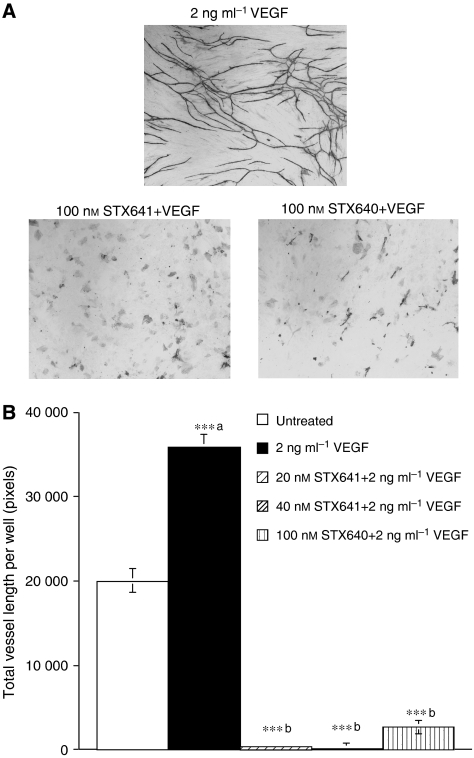
Vessel formation in an *in vitro* model of angiogenesis. (**A**) Representative high-resolution scans showing the effects of the indicated compounds on vessel formation by co-cultures of endothelial cells and fibroblasts. Cells were exposed to compounds for 11 days, and the tubules were stained using an antibody for CD31. (**B**) Co-cultures of endothelial cells and fibroblasts were exposed to compounds for 11 days. The tubules were stained using an antibody for CD31 and quantified by high-resolution scanning and subsequent image processing to quantify the number of pixels per well representing tubules. Values are means of triplicate determinations: bars; s.d. ^***a^*P*<0.001 *vs* control and ^***b^*P*<0.001 *vs* VEGF.

The representative high-resolution scans of the wells clearly show that both 100 nM STX641 and STX640 inhibit VEGF-stimulated vessel formation (Figure 4A). Quantification of the scans, using a previously validated method (Newman *et al*, 2004), shows that STX641 at both 20 and 40 nM completely blocked all tubule formation (Figure 4B). In contrast, STX140 does not completely inhibit at 20 nM (data not shown), although it does give complete inhibition at 100 nM (Newman *et al*, 2004). The nonsulphamoylated compound STX640 was not active at either 20 or 40 nM (data not shown), but did inhibit tubule formation by 92% ±2 at 100 nM (Figure 4B).

### Xenograft studies

**Figure 5:**
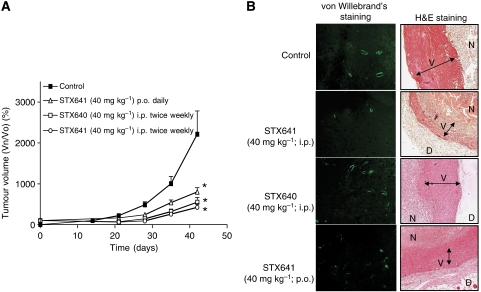
MDA-MB-231 xenograft studies. (**A**) STX641 (40 mg kg^−1^, p.o. and i.p.) and STX640 (40 mg kg^−1^ i.p.) inhibited MDA-MB-231 xenograft tumour growth *in vivo*. Data represent mean±s.e.m. (*n*=5). ^*^P<0.05 when compared to vehicle control (10% THF/90% PG; p.o.). (**B**) The effect of compounds on tumour histology and vasculature. Von Willebrand's staining of blood vessels indicated that the vehicle tumours were not greatly vascularised (**A**). However, administration of STX641 via either p.o. or i.p. caused a decrease in blood vessels. STX640 did not affect the vasculature of the tumours. Magnification × 200. In H&E stained tumour sections (**B**), three distinct areas were observed. A necrotic centre (N) surrounded by a section of viable tumour rim (V) with an outer dermis layer (D). The tumours of the vehicle-treated animals had a generally large tumour rim. This was smaller in the compound-treated groups, where STX641 caused a smaller rim than STX640. Magnification × 100.

The hormone-independent MDA-MB-231 breast xenograft model is a very robust and widely used model for the assessment of microtubule disruptors. Therefore, this model was initially used to assess the efficacy of STX641 and STX640 *in vivo*. Tumours in untreated mice increased by over 2000% in size (2215%±565 s.e.m.) over the study period; in contrast, tumours in mice treated with STX640 (40 mg kg^−1^ i.p.; twice weekly) or STX641 (40 mg kg^−1^ i.p.; twice weekly) were significantly smaller (*P*<0.001) and had only increased in size by 550% ±111 s.e. and 424%±90 s.e., respectively. Daily oral dosing with STX641 (40 mg kg^−1^ p.o.; daily) was equally as efficacious as the twice weekly i.p. dosing regime: tumours increased in size by 803% ±101 s.e. (Figure 5A). No toxicity was associated with any regime (data not shown).

To assess the *in vivo* anti-angiogenic activity of these compounds 6 μm sections of paraffin-embedded tumour were cut and stained for the endothelial-specific cell marker, von Willebrand's factor. STX641, both p.o. and i.p., significantly reduced the number of endothelial cells in tumour sections from treated mice relative to those in tumour sections from untreated mice (Figure 5B). H&E staining (Figure 5C) revealed three distinct layers, a necrotic tumour centre (N) surrounded by a healthy, viable rim (V) with a further layer of dermis (D). The tumours taken from the vehicle-treated animals generally demonstrated a larger tumour rim compared to the STX641- and STX640-treated mice.

**Figure 6:**
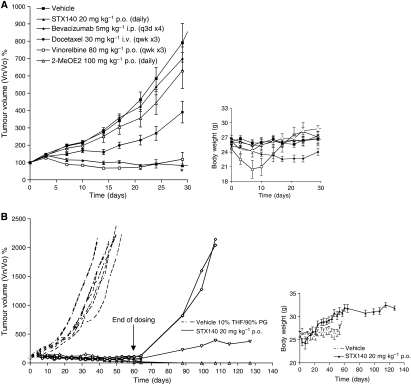
PC-3 xenograft studies. (**A**) STX140 (20 mg kg^−1^ p.o.; daily) was tested against a range of clinical and preclinical compounds in the hormone-independent PC-3 xenograft model: vehicle (10% THF/90% PG; p.o.), 2-MeOE2 (100 mg kg^−1^ p.o.; daily), bevacizumab (5 mg kg^−1^ i.p.; q3d × 3), docetaxel (i.v.; qwk × 3) and vinorelbine (80 mg kg^−1^ p.o.; qwk × 3). Data represent mean±s.e.m. (*n*=8). ^*^P<0.05 when compared to tumour starting volume. Animal weights taken over the 28-day study (inset). (**B**) Individual tumour data for the vehicle (10% THF/90% PG) and STX140 (20 mg kg^−1^ p.o.; daily) arms for the 60-day dosing schedule and the subsequent 67-day follow-up. When tumours exceeded approximately 2000 mm^3^, the animals were euthanised. Animal weights taken over the 127-day study (inset).

Despite its greater efficacy *in vitro* (Table 1), STX641 was not as potent as STX140 *in vivo*. STX641 was efficacious in the MDA-MB-231 xenograft model at 40 mg kg^−1^ p.o. (Figure 5A), whereas STX140 showed significantly greater efficacy at 20 mg kg^−1^ p.o. in a hormone-independent xenograft model (Ireson *et al*, 2004). On the basis of these observations, STX140 was selected for evaluation in the hormone-independent prostate PC-3 cell line against a panel of preclinical and clinical comparator compounds (Figure 6A). STX140 (20 mg kg^−1^ p.o.; daily) caused significant regression (*P*<0.05) after 28 days relative to the starting volumes of the tumours. No other compound caused significant tumour regression at day 28. Vinorelbine (80 mg kg^−1^ p.o.; qwk × 3) prevented tumour growth, however significant weight loss was observed (Figure 6A) and two animals died during the study, indicative of significant toxicity. Docetaxel (30 mg kg^−1^ i.v.; qwk × 3) significantly (*P*<0.001) slowed tumour growth, but tumour size still increased four-fold with docetaxel dosing. Both bevacizumab (5 mg kg^−1^ i.p.; q3d × 4) and 2-MeOE2 (100 mg kg^−1^ p.o.; daily) had no significant effect on tumour growth relative to vehicle (10% THF/90% propylene glycol (PG)).

Extended (28 days+32 days=60 days) daily dosing with STX140 (20 mg kg^−1^ p.o.) led to 5/8 tumours regressing and 3/8 not increasing in size. After the cessation of dosing on day 60, the growth of 5/8 tumours was followed for a further 67 days (Figure 6B). Of these tumours, 2/5 disappeared by day 88 and were still undetectable at the end of study (Δ), 1/5 progressed slowly and had only increased in volume by threefold at day 127 relative to day 0 (∇). The remaining two tumours grew rapidly and the animals were taken off study on day 107 (◊). No significant weight loss was seen during the 60-day dosing schedule or the 67-day follow-up period (Figure 6B, inset). All the untreated tumours (○) grew rapidly, and the animals were all taken off study by day 50 as the tumours had exceeded 2000 mm^3^.

## DISCUSSION

Recent studies by Liu *et al* (2005) and our group (Newman *et al*, 2006) have highlighted the limitations of 2-MeOE2 as a therapeutic agent due to its potential for metabolism and subsequent inactivation by 17*β*-HSD type 2 at the C-17 position in the D-ring. This suggested a need for protective modification of 2-MeOE2 and other similar compounds at this position. Therefore, the effects of further C-17 modifications to the A-ring-modified sulphamoylated compounds previously investigated by our group (MacCarthy-Morrogh *et al*, 2000), and, in addition, the effects of C-17 modification on the activity of a related nonsulphamoylated compound have been explored.

*In vitro* studies have shown STX140 to be a potent inhibitor of proliferation in a wide range of tumour cell lines, with IC_50_ values approximately 10-fold less than 2-MeOE2 (Day *et al*, 2003; Newman *et al*, 2004). In this study, the replacement of the 17-*O*-sulphamate of STX140 with a cyanomethyl group significantly enhances the anti-proliferative activity. Furthermore, the nonsulphamoylated C-17 cyanomethyl compound, STX640, was approximately 10 times more potent than 2-MeOE2. These results indicate that a C-17 cyanomethyl group is able to impart anti-proliferative activity on this class of compounds, even in the absence of a 3-*O*-sulphamate group. All three compounds caused irreversible inhibition of cell proliferation, with only minimal proliferation in STX140- and STX640-treated PC-3 cells after compound removal. Thus, further demonstrating the importance of the C-17 cyanomethyl substitution, as previous studies (MacCarthy-Morrogh *et al*, 2000; Day *et al*, 2003; Raobaikady *et al*, 2005) have shown the nonsulphamoylated 2-MeOE2 to be a reversible inhibitor of cell proliferation.

To further characterise the C-17 cyanomethyl derivatives, their effects on the cell cycle were assessed and compared with those of STX140 in the PC-3 cells. All compounds caused G_2_/M cell cycle arrest and subsequent apoptosis, there was no significant difference in efficacy between the compounds. The potency of STX640 in this assay again emphasises the value of adding a C-17 cyanomethyl group to this class of compound. The proliferation, cell cycle and apoptosis data were further supported by the improved activity of STX641 *vs* STX140 and STX640 *vs* 2-MeOE2 in the inhibition of paclitaxel-stimulated tubulin polymerisation. This highlights the potential tubulin interaction role of the C-17 cyanomethyl and possibly explains the better efficacy of C-17 cyanomethyl compounds.

2-MeOE2, STX641, STX640 and STX140 inhibit the proliferation of HUVECs, and this is an indicator of potential anti-angiogenic activity. Using a previously described (Newman *et al*, 2004) *in vitro* co-culture model of angiogenesis, the anti-angiogenic potential of STX641 and STX640 was assessed. Both compounds were potent inhibitors of VEGF-stimulated angiogenesis in this model, with STX641 being the most potent compound. In this same assay, 100 nM 2-MeOE2 had no effect (Newman *et al*, 2004); in contrast, the nonsulphamoylated STX640 reduced angiogenesis by 90%. The mechanisms by which 2-MeOE2 and related sulphamoylated derivatives inhibit angiogenesis have yet to be fully resolved. One likely explanation arises from the observations that both 2-MeOE2 and related sulphamoylated derivatives cause G_2_/M arrest in endothelial cells (Lakhani *et al*, 2003; Newman *et al*, 2004), inhibit the proliferation of endothelial cells, and the sulphamate derivatives have been shown to induce Bcl-2 phosphorylation and apoptosis in endothelial cells (Newman *et al*, 2004). Thus, it seems likely that in a growing tumour, the endothelial cells will be rapidly dividing to meet the tumour's demand for angiogenesis, and that this class of compound may act as a classic microtubule disruptor, inducing apoptosis in rapidly dividing cells whether they are of tumour or endothelial origin. This concept is currently being evaluated for many ‘traditional’ chemotherapeutic compounds, with regular, low (metronomic) dosing being used in an effort to maximise the anti-angiogenic action and maintain the anti-tumour activity (Vacca *et al*, 1999; Hanahan *et al*, 2000; Bertolini *et al*, 2003).

The potential anti-angiogenic activity of STX641 identified *in vitro* was confirmed by the reduction in staining for von Willebrand's factor, an endothelial cell-specific marker, observed *in vivo*. Surprisingly, the significantly improved efficacy of STX641 in comparison to STX140 *in vitro* did not translate to significant improvements *in vivo*; in fact, comparison with STX641 shows STX140 to be more potent at inhibiting both tumour growth (Ireson *et al*, 2004) and *in vivo* angiogenesis (Chander *et al*, 2007). The observation that twice weekly i.p. administration of STX641 was equipotent to daily p.o. administration at the same dose (40 mg kg^−1^) strongly indicates that STX641 may not be as readily orally bioavailable as STX140 with the formulation used. For these reasons, STX140 was selected for further *in vivo* studies in the hormone-independent PC-3 prostate xenograft model. To fully assess the potential of STX641, further formulation and PK studies need to be undertaken. To evaluate STX140, it was tested against a range of clinically relevant drugs. Both STX140 (20 mg kg^−1^ p.o.) and vinorelbine (80 mg kg^−1^ p.o.) completely inhibited tumour growth over a 28-day period. However, in contrast to vinorelbine, STX140 did not cause any significant weight loss. Docetaxel, which is commonly used to treat hormone-independent prostate cancer, only slowed the rate of tumour growth by about 50%. This, taken in conjunction with its associated toxicity and lack of oral bioavailability in the clinic, indicates that there is significant room for improvement in the treatment of advanced prostate cancer.

Bevacizumab inhibits VEGF and prevents its binding to the VEGFR2 and stimulating angiogenesis. In this study, bevacizumab had no significant effect on tumour growth. The role of VEGF in sustaining the growth of PC-3 xenografts is difficult to ascertain as Takei *et al* (2004) showed siRNA against VEGF could reduce xenograft growth, but Dev *et al* (2004) showed that PC-3 cells expressed little VEGF and were not sensitive to a VEGFR2 inhibitor. The poor efficacy of 2-MeOE2 *in vivo* was further highlighted by its lack of activity in the PC-3 model, despite daily dosing at 100 mg kg^−1^ p.o. Data generated from this study strongly favour STX140 as a potential clinical candidate for hormone-independent prostate cancer. Further studies could be undertaken using more sophisticated *in vivo* models, such as orthotopic or metastatic prostate models to expand upon these results. The use of STX140 was further supported by extended dosing studies, in which STX140 could be administered daily (20 mg kg^−1^ p.o.) for 60 days with no toxicity being observed. This prolonged dosing schedule produced remarkable results with complete or partial responses seen in 8/8 tumours. Furthermore, no tumours could be detected in the 2/8 complete responses 67 days after the cessation of dosing.

This study demonstrates for the first time that addition of a C-17 cyanomethyl group significantly enhances the *in vitro* and *in vivo* efficacy of 2-MeOE2-like compounds. The cyanomethyl-substituted compounds STX640 and STX641 disrupt microtubule dynamics leading to apoptosis, and both are potent (nM range) inhibitors of *in vitro* angiogenesis. *In vivo*, in the hormone-independent MDA-MB-231 breast xenograft model, both compounds significantly inhibit tumour growth. However, *in vivo*, neither compound is as potent as STX140, which was shown to be superior to a range of clinical and developmental compounds, including docetaxel and bevacizumab, in the PC-3 hormone-independent xenograft model. Finally, data presented in this study suggest that STX140 may allow for regular, oral, prolonged dosing regimes with minimal toxicity and without the need to compromise the dose used. Thus, STX140 could be regarded as a new generation of oral microtubule disruptor, which has direct anti-tumour activity and reduces tumour vasculature at one optimal dose. These properties suggest that this compound could be efficacious in both hormone-independent prostate and breast cancer. STX140 is envisaged to enter the clinic in 2008.

## Figures and Tables

**Table 1:** The effect of 2-substituted, C-17-modified oestrogens on cell proliferation in a panel a cell lines (IC_50_ values in nM)

**Cell line**	**Origin**	**STX641**	**STX640**	**STX140^a^**
PC-3	Prostate AR−ve	50a,b	430c	270
MDA-MB-231^b^	Breast ER−ve	80a,b	320NS	290
LNCaP	Prostate AR+ve	60a,b	320NS	260
MCF-7	Breast ER+ve	150a,b	320c	250
A2780	Ovarian ER−ve	90a,b	330NS	280
HUVEC	Endothelial	30NS,b	30c	44

HUVEC=human umbilical vein endothelial cell.

Statistics: a, STX641 *vs* STX640 *P*<0.01; b, STX641 *vs* STX140 *P*<0.01; c, STX640 *vs* STX140 *P*<0.05; NS, not significant.

a Data presented for comparative purposes previously published by Newman *et al* (2004) and Day *et al* (2003).

b Data presented for comparative purposes previously published by Utsumi *et al* (2005).
